# Effects of Surface Epitope Coverage on the Sensitivity of Displacement Assays that Employ Modified Nanoparticles: Using Bisphenol A as a Model Analyte

**DOI:** 10.3390/bios6030043

**Published:** 2016-08-08

**Authors:** Yang Lu, Joshua Richard Peterson, Erwann Luais, John Justin Gooding, Nanju Alice Lee

**Affiliations:** 1 School of Chemical Engineering, University of New South Wales, Sydney, NSW 2052, Australia; luyang@tust.edu.cn; 2 School of Chemistry, University of New South Wales, Sydney, NSW 2052, Australia; peterson.j@unsw.edu.au (J.R.P.); erwann.luais@univ-tours.fr (E.L.); 3 School of Chemistry and Australian Centre for NanoMedicine, University of New South Wales, Sydney, NSW 2052, Australia; 4 School of Chemical Engineering and ARC Training Centre for Advanced Technologies in Food Manufacture, University of New South Wales, Sydney, NSW 2052, Australia

**Keywords:** bisphenol A, gold nanoparticles, displacement ELISA, surface epitope density

## Abstract

With the ever-increasing use of nanoparticles in immunosensors, a fundamental study on the effect of epitope density is presented herein, with a small molecule epitope, on the performance of the displacement assay format in an enzyme-linked immunosorbent assay (ELISA). Thiolated bisphenol A (BPA) functionalized gold nanoparticles (cysBPAv-AuNPs) and specific anti-BPA antibodies are employed for this purpose. It is shown that the displacement of cysBPAv-AuNPs bound to the immobilized antibodies was influenced by both the avidity of bound cysBPAv-AuNPs and the concentration of free BPA to displace it. The importance of surface epitope density was that it changed the number of epitopes in close proximity to the antibody-binding site. This then influenced the avidity of cysBPAv-AuNPs bound to the immobilized antibody. Furthermore, the molar epitope concentration in an assay appears to affect the degree of antibody binding site saturation. Controlling surface epitope density of the functionalized nanoparticles and molar epitope concentration in an assay leads to a decrease of the concentration of free BPA required to displace the bound cysBPAv-AuNP, and hence better assay performance with regards to the D_50_ value and dynamic range in the displacement assay.

## 1. Introduction

The fabrication of nanoparticles and the exploration of their properties have been increasingly utilized in many branches of science, including chemistry, biology, physics, and engineering [1,2,3,4,5]. A particular focus on these studies has been the unique size-dependent electrical and optical properties relative to the equivalent bulk materials [6,7,8]. Furthermore, nanoparticles can be functionalized with both biological molecules and small organic compounds to allow their application as biomarker, drug delivery vehicles, and biosensor receptors [9,10,11]. For instance, in the study by Liu et al. [10], gold nanoparticles were functionalized with both antibodies and diazonium salts to fabricate a robust and versatile interface for the electrochemical sensing of botulinum neurotoxin type A. The potential of functionalized nanoparticles in immunodiagnostic assays and immunosensors for small molecules is that they could replace proteins as the platform upon which small molecule epitopes are attached. This is an attractive notion, as surface functionalization of nanoparticles confers far greater control over epitope density—and potentially assay performance—than protein analogues. We have recently begun to explore the impact of nanoparticle surface epitope density on immunoassay performance with a view to developing better-performing immunosensors [12]. Here we turn our attention to asking questions regarding the effects of the density of nanoparticle-bound epitopes on displacement assays, rather than competitive inhibition assays, as explored previously [12]. This is because displacement assays are much more compatible with the development of reagentless immunosensors that do not require washing and rinsing steps, as all of the reagents are surface bound [13,14,15].

A displacement immunosensor is one where the presence of an analyte causes an antibody to be displaced from the sensing surface with a concomitant signal change. Despite their potential, however, displacement assays are notoriously difficult to configure. A major part of the challenge in configuring a displacement immunosensor comes down to poor control of the interaction between the biomolecule/antibody and the sensing surface [16]. We investigated the impact of the affinity between the surface-bound epitope and the antibody (IgM) on the sensitivity and specificity of a displacement electrochemical immunosensor [16]. In this study, the sensitivity of a displacement assay is confirmed to be determined by the affinity constant of the epitope/antibody [16]. The lower the affinity of the antibody for the surface epitope is, the higher the sensitivity that could be achieved, but potentially at the cost of selectivity [16].

At present, little is known about the relationship between surface epitope coverage (equivalent to epitope density in hapten–protein conjugates) and the related parameters on nanoparticles, and assay parameters in a displacement assay setting. Nanoparticles provide an exquisite surface control for an antibody-immobilized sensor surface, which then affords considerable control over assay parameters. Furthermore, the plasmonic nature of metal nanoparticles can act as labels to give signals. The binding affinity of the functionalized nanoparticles will have a significant impact on the sensitivity of the displacement immunosensor [16].

Using gold nanoparticles modified with small organic epitopes for a surface affinity interaction with an antibody as a model, we could explore questions about displacement-based biosensing systems, such as which factors affect the binding affinity of the functionalized nanoparticles and how these factors affect assay parameters such as sensitivity, dynamic range, and limit of detection. Following our previous papers on the conventional immunoassay [17] and nanoparticle competitive immunoassay [12], this paper uses bisphenol A (BPA) as a model analyte and focuses on the evaluation of thiolated BPA-functionalized gold nanoparticles (cysBPAv-AuNPs) with different surface epitope coverage and the related factors via a displacement assay devised with two antibodies.

## 2. Materials and Methods

### 2.1. Materials

Hydrogen tetrachloroaurate(III) hydrate, sodium borohydride, 4,4-bis(4-hydroxyphenyl)valeric acid (BPA-valeric acid), *N*-hydroxysuccinimide (NHS) and *N*,*N*′-dicyclohexylcarbodiimide (DCC), thimerosal, dimethyl sulfoxide (DMSO), bovine serum albumin (BSA), biotin, avidin-horseradish peroxidase (HRP), 3,3′,5,5′-tetramethylbenzidine (TMB), and Tween-20 were purchased from Sigma-Aldrich (Sydney, Australia). Absolute ethanol (EtOH), methanol (MeOH), dimethylformamide (DMF), tri-sodium citrate, sulphuric acid, hydrochloric acid, nitric acid, and hydrogen peroxide were obtained from Ajax Finechem (Sydney, Australia). Soybean protein (SBP) was purchased from Nature’s Way (Sydney, Australia). For the preparation of buffers, chemicals used were sourced from either BDH Chemicals (Melbourne, Australia) or Ajax Finechem (Sydney, Australia). Maxisorp polystyrene 96-well plates were obtained from Nunc (Roskilde, Denmark). Deionized water was prepared from a Millipore Milli-Q Academic system (18.2 MΩ·cm). Reverse osmosis (RO) water (17.6 MΩ·cm) of 99.0% purity was sourced from Sartorius Arium 61316/611VF. The preparation of cysteamine-BPA-valerate (cysBPAv), AuNP, the rabbit anti-BPA antibody (Ab-BPA-V2#4), and the conjugation of an antibody and biotin (Ab-BPA-V2#4-biotin) were described in the previous publications [12,17,18].

### 2.2. Instrumentation

An ELISA plate reader (SpectroMax M2) was from Molecular Devices (Sunnyvale, CA, USA). Centrifugation was performed using a Sigma 1-14 laboratory tabletop microcentrifuge. UV-Vis measurements were performed on a Varian Cary 50 Bio UV-Visible spectrophotometer.

### 2.3. Modification of AuNPs

The detailed synthesis of cysBPAv and the chemical environments of gold nanoparticles before and after functionalization with the cysBPAv analyzed by X-ray photoelectron spectroscopy has been described in [12,18]. Based on the modification of AuNPs described previously [12], different volumes (13, 15, 20, 30, and 60 µL) of 1 mmol·L^−1^ of cysBPAv in EtOH was added to 1 mL of AuNPs solution, and the mixture was allowed to stand at room temperature overnight in the dark to synthesize five batches of AuNPs with different surface epitope coverage, denoted as cysBPAv-AuNP-163, cysBPAv-AuNP-190, cysBPAv-AuNP-265, cysBPAv-AuNP-396, and cysBPAv-AuNP-801, respectively [12]. The calculation of epitope coverage (i.e., the number of cysBPAv ligand conjugated per nanoparticle) was described previously [12] and depicted in parentheses (e.g., cysBPAv-AuNP-163 corresponds to particle cysBPAv-AuNP with 163 conjugated ligands). The epitope coverage of cysBPAv-AuNP-163, cysBPAv-AuNP-190, cysBPAv-AuNP-265, cysBPAv-AuNP-396, and cysBPAv-AuNP-801 was calculated in the previous paper and presented as 3.0 × 10^−10^, 3.6 × 10^−10^, 4.9 × 10^−10^, 7.4 × 10^−10^, and 15.0 × 10^−10^ mol·cm^−2^, respectively [12]. After centrifugation (16,163× *g* for 15 min) of the cysBPAv-AuNP, the pellet of nanoparticles was resuspended in 100 µL EtOH and made up to 1 mL with Milli-Q water.

### 2.4. Preparation of BPA Standard

A stock solution of BPA in MeOH (1 × 10^6^ µg·L^−1^) was prepared in a volumetric flask. Aliquots of the stock solution in amber glass vials were stored at 4 °C. Working standard solutions (0.51–10,000 µg·L^−1^) were prepared by diluting the stock solution in 10% EtOH in phosphate buffered saline (PBS) just before use.

### 2.5. ELISA via the Displacement Format (d-ELISA)

**Figure 1:**
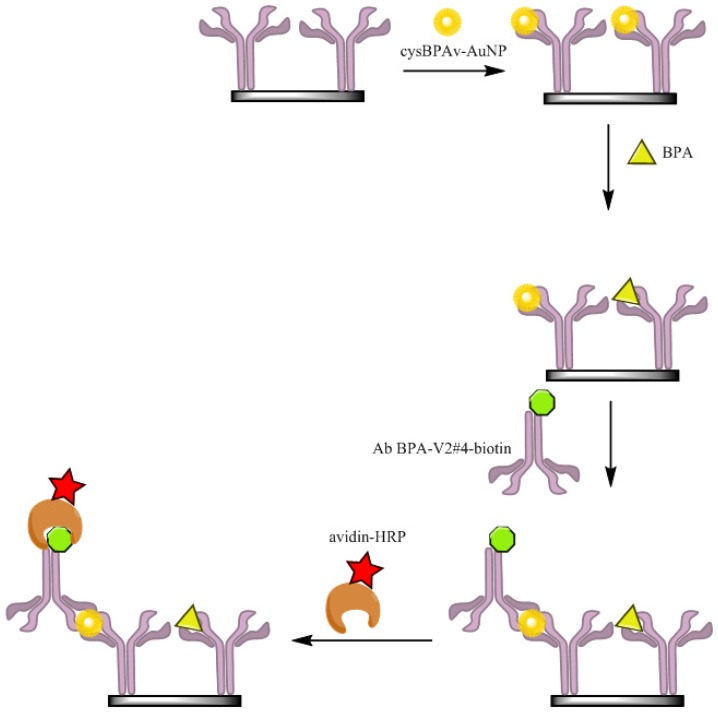
The configuration of the displacement-ELISA (d-ELISA). Ab BPA-V2#4-biotin: anti-BPA antibody–biotin (green) conjugate; Avidin-HRP: avidin (brown)-horseradish peroxidase (red); cysBPAv-AuNP: thiolated bisphenol A (BPA)-functionalized gold nanoparticles (yellow).

A nanoparticle displacement immunoassay using two rabbit anti BPA-valerate antibodies performing capturing and detection was devised to study the effects of surface epitope coverage on assay performance. In this assay, the anti-BPA antibody (Ab-BPA-V2#4) as the capture antibody was immobilized at 0.1 µg·well^−1^. After washing with 0.05% Tween-20, 1% SBP–PBS was incubated for 1 h. Thereafter, cysBPAv-AuNPs in 10% EtOH/PBS was added to their respective wells and allowed to incubate for 15 min. Then, BPA standards prepared in 10% EtOH in PBS were added to the respective wells and incubated (with gentle shaking to minimize AuNP settling) for 30, 60, and 120 min. After washing the plates, the microwells were incubated with 1.2 µg·mL^−1^ anti-BPA antibody-biotin conjugate (Ab-BPA-V2#4–biotin) as the detection antibody for 1 h. A further three washes were performed, and then the surfaces were incubated in avidin-HRP for 30 min. The wells were washed five times with 0.05% Tween-20, and a TMB substrate solution was added to develop the color. After 20 min, 50 µL of 1.25 mol·L^−1^ sulphuric acid was added to the wells to stop further color development. Then measurement of the absorbance at 450 nm was conducted using a microplate reader (Figure 1).

### 2.6. Percent Relative Saturation (%RS)

%RS was calculated by the following equation:

$$\%\text{RS}=\left(\frac{A_{\text{BPA}}}{A_{c}}\right)\times 100$$

where *A*_c_ = absorbance value of control, and *A*_BPA_ = absorbance value with free BPA as a standard. The *A*_c_ is determined as the maximum absorbance at which no displacement of the bound cysBPAv-AuNPs is induced. %RS curve is a dose–response curve of the displacement assay. D_50_ is defined as the concentration of BPA that displaces 50% of bound nanoparticles.

## 3. Results and Discussion

In order to study the influence of surface epitope coverage and the related parameters on antibody–surface epitope binding in a displacement assay and subsequent assay sensitivity, a nanoparticle displacement assay using microwell plates was devised. This assay (using a microwell plate) allowed us to modify and optimize experimental conditions quickly and easily, and the experiments were conducted with multiple replicates for better statistical analysis. The displacement between a BPA-modified nanoparticle and free BPA for a BPA-specific antibody is represented by the following equation, based on the law of mass action.

$$\text{Ab}+{\text{BPA}}^{*}\;\begin{array}{c} k_{a1}\\ ↔\\ k_{d1} \end{array}\;\left[\text{Ab}:{\text{BPA}}^{*}\right]+\text{BPA}\begin{array}{c} k_{a2}\\ ↔\\ k_{d2} \end{array}\left[\text{Ab}:\text{BPA}\right]+{\text{BPA}}^{*}$$

where Ab is the BPA-specific antibody immobilized on a solid phase, BPA* is BPA-modified nanoparticles, [Ab:BPA*] is the complex of immobilized antibody and BPA-modified nanoparticles, BPA is the unlabeled BPA added to the assay to displace BPA*, [Ab:BPA] is the complex of immobilized antibody and unlabeled BPA, and BPA* (on the far right) is the BPA-modified nanoparticles displaced from the immobilized antibody. The configuration of the nanoparticle-based displacement ELISA—henceforth referred to as d-ELISA—is shown in Figure 1. The following assumption was made to simplify the affinity interaction between reagents occurring in an assay. We assumed that for the majority of the antibody molecules, only one BPA-modified nanoparticle would bind to one binding site of the immobilized antibody due to the space constraints (i.e., valence n = 1). We also made the assumption that the affinity of the BPA antibody for the cysBPAv-AuNPs is sufficiently strong so that dissociation of the antibody–cysBPAv-AuNPs complex (in the absence of BPA) is negligible during the assay. This assumption was made based on the consistent results obtained from the control wells. We also made the assumption that the heterogeneous nature of the polyclonal antibody used in this study was consistent between the reaction wells, and would not affect the apparent affinity with the AuNPs. From the above equation representing the displacement assay, K_a2_, therefore, needs to be higher than K_a1_ for effective displacement to occur.

Using the devised two-antibody nanoparticle displacement assay with the avidin–biotin system to enhance color development, the following sets of experiments were conducted to evaluate how each of the key factors related to the epitope-functionalized nanoparticles influence the assay performance in a displacement format. The factors investigated were the surface epitope coverage, molar concentration of particles, and molar concentration of epitope in an assay. In each set of experiments below, one of the factors was kept constant to allow the other two factors to vary.

### 3.1. d-ELISA with Different Displacement Time

Displacement of a surface-bound small molecule epitope from an antibody by an analyte is a time-dependent process influenced by the binding affinity for the epitope [16]. To investigate the effects of incubation time of free BPA on the displacement of the bound cysBPAv-AuNPs in the nanoparticle d-ELISA, and to establish the optimal assay conditions for the subsequent experiments, varying BPA incubation times (30, 60, and 120 min) over a broad range of BPA concentrations were studied with cysBPAv-AuNP-265. We chose cysBPAv-AuNP-265 because it showed sufficiently strong and stable binding between the cysBPAv-AuNPs and the immobilized antibody in the preliminary study (not shown).

**Figure 2:**
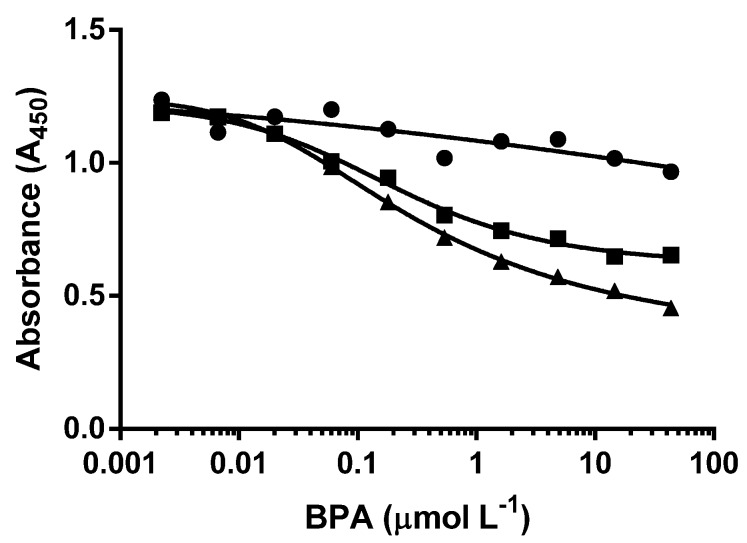
Displacement of cysBPAv-AuNP-265 bound to Ab-BPA-V2#4 by free BPA with different incubation times: ● = 30 min, ■ = 60 min, ▲ = 120 min. CysBPAv-AuNP-265 corresponds to particle cysBPAv-AuNP with 265 conjugated ligands.

The displacement of bound cysBPAv-AuNPs by the free BPA was evident by the decrease in absorbance values of the testing wells in a concentration-dependent manner (Figure 2). In the 30 min incubation time, little and inconsistent displacement was observed, even at 10,000 µg·L^−1^. Greater displacement of the bound cysBPAv-AuNPs with greater consistency was observed for 60 min and 120 min incubations. These results confirmed that the displacement of bound cysBPAv-AuNPs by the free BPA was a time-dependent process, influenced by the affinity of the antibody for the surface epitope. The time taken for the cysBPAv-AuNPs to displace the antibody binding sites was substantially longer than anticipated. This was likely to be due to the hapten used to prepare epitope-functionalized nanoparticles, which was homologous to the hapten used for raising the specific antibodies, meaning the affinity of the antibody for the surface-bound epitope was high, and hence displacement was difficult. The incubation time for more consistent displacement was 120 min for this assay, and was chosen to conduct the next three sets of experiments.

### 3.2. d-ELISA with Different Molar Concentrations of cysBPAv-AuNP-265

**Figure 3:**
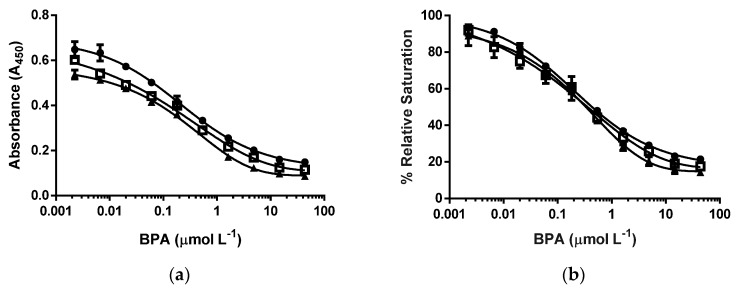
d-ELISA under the different molar concentrations of cysBPAv-AuNP-265. (**a**) Absorbance curve, and (**b**) % relative saturation. ● = the molar concentration of cysBPAv-AuNP-265 was 6.6 × 10^-10^ mol·L^−1^ (the concentration of epitope in an assay: 1.7 × 10^−7^ mol·L^−1^); □ = the molar concentration of cysBPAv-AuNP-265 was 3.3 × 10^−10^ mol·L^−1^ (the concentration of epitope in an assay: 9.0 × 10^−8^ mol·L^−1^); ▲ = the molar concentration of cysBPAv-AuNP-265 was 1.3 × 10^−10^ mol·L^−1^ (the concentration of epitope in an assay: 3.0 × 10^−8^ mol·L^−1^).

The next set of experiments was conducted to compare the molar concentration of nanoparticle and the related molar concentration of epitope in an assay of the displacement of bound nanoparticles using cysBPAv-AuNP-265 as a fixed nanoparticle-bound epitope. A molar concentration of epitope in an assay was defined as the total concentration of epitope in an assay solution, and in this experiment, the molar concentration of epitope in an assay was kept between 1.3 × 10^−10^ mol·L^−1^ and 6.6 × 10^−10^ mol·L^−1^ (five-fold difference). Lowering the molar concentration of particle—and concurrently the amount of surface epitope—decreased the total bound antibody in an assay, as observed by the decrease in absorbance. Note that the decrease in the absorbance was relatively small compared to the difference in the molar concentration of particles (Figure 3, Table 1). The insensitivity of the assay on the molar concentration of particles and epitope was also observed for the values of the D_50_, limit of detection, and dynamic range, all of which only decreased slightly with decreasing molar concentration of epitopes and particles.

### 3.3. d-ELISA with the Same Molar Concentration of cysBPAv-AuNP-190, cysBPAv-AuNP-396, and cysBPAv-AuNP-801

To investigate the effects of epitope coverage and molar concentration of epitope in an assay on the displacement of nanoparticles from antibody binding, the molar concentration of the particles cysBPAv-AuNP-190, cysBPAv-AuNP-396, and cysBPAv-AuNP-801 were fixed to 2.1 × 10^−10^ mol·L^−1^. Based on the estimated epitope densities on each particle type, the molar concentration of the epitope in an assay was determined to be 4.0 × 10^−8^ mol·L^−1^ for cysBPAv-AuNP-190, 8.0 × 10^−8^ mol·L^−1^ for cysBPAv-AuNP-396, and 1.7 × 10^−7^ mol·L^−1^ for cysBPAv-AuNP-801, respectively.

**Figure 4:**
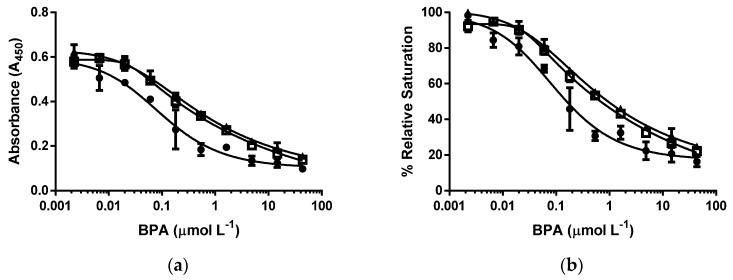
Dose–response curves of d-ELISA under the same molar concentration of cysBPAv-AuNP-190, cysBPAv-AuNP-396, and cysBPAv-AuNP-801. (**a**) Absorbance curve, and (**b**) % relative saturation. ● = the molar concentration of cysBPA-AuNP-190 was 2.1 × 10^−10^ mol·L^−1^ (the molar concentration of epitope in an assay: 4.0 × 10^−8^ mol·L^−1^); □ = the molar concentration of cysBPA-AuNP-396 was 2.1 × 10^−10^ mol·L^−1^ (the molar concentration of epitope in an assay: 8.0 × 10^−8^ mol·L^−1^); ▲ = the molar concentration of cysBPA-AuNP-801 was 2.1 × 10^−10^ mol·L^−1^ (the molar concentration of epitope in an assay: 1.6 × 10^−7^ mol·L^−1^).

All three nanoparticles showed similar relative maximum nanoparticle binding (i.e., % relative saturation), suggesting that these particles had sufficient surface epitopes to induce particle–antibody binding in the absence of the antigen (i.e., BPA). CysBPAv-AuNP-396 and cysBPAv-AuNP-801 showed no significant differences in the displacement of the bound nanoparticle by BPA (Figure 4, Table 2). Steric effects of higher epitope coverage may have limited the number of nanoparticles that could be bound to the immobilized antibody. The cysBPAv-AuNP-190 particles with lower epitope coverage showed greater displacement than the cysBPAv-AuNP-396 and cysBPAv-AuNP-801particles with higher epitope coverage. The D_50_, limit of detection, and dynamic range of cysBPAv-AuNP-190 was much lower than those of cysBPAv-AuNP-396 and cysBPAv-AuNP-801. These suggest that epitope coverage affects the avidity of surface epitope–antibody binding, and subsequent displacement of bound nanoparticles by the addition of BPA. Having lower epitope coverage is advantageous in a displacement event, provided that the initial binding of nanoparticles to the immobilized antibody is sufficiently strong and fast enough to withstand the assay procedure. The degree of nanoparticle displacement is affected by the BPA concentration added to displace the bound nanoparticles, as shown by the sigmoidal relationship in Figure 4b. Interestingly, increasing the concentration of BPA above 43.8 µmol·L^−1^ did not completely displace the bound nanoparticles, and 20%–30% cysBPAv-AuNP remained bound. This could be due to the heterogeneity of polyclonal antibody binding to BPA [19] or nonspecific adsorption of particles on the blocking protein or solid phase [14]. It is, however, unclear which of the two factors (i.e., epitope coverage and molar concentration of epitope in an assay) has the greater effect on the assay parameters, or if both factors contribute to the overall effect.

### 3.4. d-ELISA with the Same Molar Concentration of Epitope in an Assay of cysBPAv-AuNP-190, cysBPAv-AuNP-396, and cysBPAv-AuNP-801

**Figure 5:**
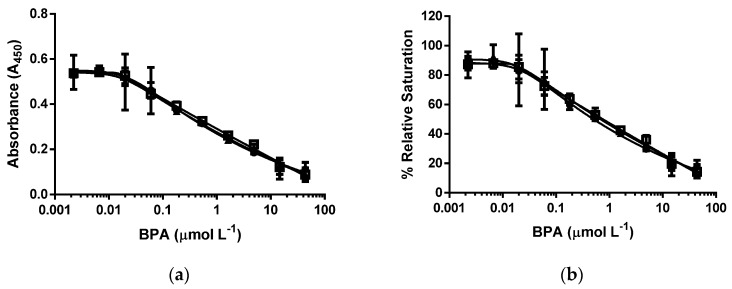
Dose–response curves of d-ELISA under the same molar concentration of epitope in an assay in cysBPAv-AuNP-190, cysBPAv-AuNP-396, and cysBPAv-AuNP-801 solutions. (**a**) Absorbance curve and (**b**) % relative saturation. ● = the molar concentration of cysBPAv-AuNP-190 was 5.3 × 10^−10^ mol·L^−1^ (the molar concentration of epitope in an assay: 1.0 × 10^−7^ mol·L^−1^); □ = the molar concentration of cysBPAv-AuNP-396 was 2.6 × 10^−10^ mol·L^−1^ (the molar concentration of epitope in an assay: 1.0 × 10^−7^ mol·L^−1^); ▲ = the molar concentration of cysBPAv-AuNP-801 was 1.3 × 10^−10^ mol·L^−1^ (the molar concentration of epitope in an assay: 1.0 × 10^−7^ mol·L^−1^).

In the third set of experiments, the molar concentration of epitope in an assay of the tested particles was kept at 1.0 × 10^−7^ mol·L^−1^ to investigate the effects of the epitope coverage and the molar concentration of particle on the displacement of bound cysBPAv-AuNPs. In this study, the nanoparticle concentrations of cysBPAv-AuNP-190, cysBPAv-AuNP-396, and cysBPAv-AuNP-801 were set at 5.3 × 10^−10^, 2.6 × 10^−10^, and 1.3 × 10^−10^ mol·L^−1^, respectively. All three %RS curves of these cysBPAv-AuNPs were overlapping each other with very similar D_50_ values and limits of detection. For the same total concentration of epitope, the epitope coverage per particle and molar concentration of particle in an assay did not influence the displacement of bound nanoparticles (Figure 5, Table 3). The limit of detection and dynamic range of all these three sets of cysBPAv-AuNPs were similar. It was noted that the nanoparticles in the range of molar concentrations studied in this experiment did not show any physical interference. These results were also consistent with our previous study [12] and further confirmed AuNPs’ function as the epitope carriers and that they did not participate in the assay.

## 4. Conclusions

The interactions of epitope-functionalized nanoparticles and specific antibodies in a displacement assay were studied using a two-antibody displacement assay devised for this study. The effects of three key assay parameters relating to surface epitopes were studied: (1) surface epitope coverage per particle, which represents micro-environment between surface epitopes and antibody binding sites when they are in close proximity; and (2) molar concentration of particles and (3) molar concentration of epitope in an assay, both of which represent the overall antibody–epitope interaction in an assay.

All of the conditions tested in the three sets of experiments with a decreasing molar concentration of particles or surface epitope coverage per particle led to decreasing D_50_ and dynamic ranges. With the same molar concentration of epitope in an assay, most of the experimental conditions tested resulted in saturation of antibody binding sites and similar binding avidity. Displacement of the bound nanoparticles, therefore, required similar concentrations of free BPA. Only with one condition, with lower surface epitope coverage (cysBPAv-AuNP-190 with 3.6 × 10^−10^ mol·cm^−2^), lower molar particle concentration (2.1 × 10^−10^ mol·L^−1^), and lower molar concentration epitopes (4.0 × 10^−8^ mol·L^−1^), resulted in a significant decrease in the D_50_ and dynamic range.

These results have led us to propose the following conclusion. Surface epitope coverage is an important factor, as it influences the amount of local epitope in close proximity to the antibody binding site on the solid–liquid interface. This local epitope concentration then influences the overall avidity of the cysBPAv-AuNPs bound to the immobilized antibody. Displacement of bound cysBPAv-AuNPs is influenced by apparent avidity of bound cysBPAv-AuNPs. Molar epitope concentration in an assay appears to have an effect on the degree of saturation of antibody binding site, but not the displacement of bound nanoparticles.

Despite the potential shortcomings of the system used, our study provides insights into key factors that may influence the sensitivity of the displacement assay and design of epitope-functionalized nanoparticles as a carrier for sensor platform fabrication. We recommend lower surface epitope coverage for a displacement-type assay, but it needs to be balanced with avidity of the surface epitope (on nanoparticles) binding to the antibody. Controlling surface epitope coverage as the key factor and total epitope concentration in an assay to a lesser extent would provide an effective means to decrease D_50_ and achieve an ideal dynamic range for a displacement assay.

## Figures and Tables

**Table 1:** Displacement assay parameters derived from Figure 3.

Surface Epitope	265 (●)	265 (□)	265 (▲)
Particle number (×10^−10^ mol·L^−1^)	6.6	3.3	1.3
Molar concentration of epitope (×10^−7^ mol·L^−1^)	1.7	0.9	0.3
D_50_ (µmol·L^−1^)	0.5	0.4	0.3
Limit of Detection (µmol·L^−1^)	0.03	0.02	0.01
Dynamic range (µmol·L^−1^)	0.03–42.7	0.02–12.0	0.01–4.0

**Table 2:** Displacement assay parameters derived from Figure 4.

Surface Epitope	190 (●)	396 (□)	801 (▲)
Particle number (×10^−10^ mol·L^−1^)	2.1	2.1	2.1
Molar concentration of epitope (×10^−7^ mol·L^−1^)	0.4	0.8	1.6
D_50_ (µmol·L^−1^)	0.2	0.7	0.7
Limit of Detection (µmol·L^−1^)	0.01	0.02	0.02
Dynamic range (µmol·L^−1^)	0.01–2.3	0.02–20.0	0.02–39.8

**Table 3:** Displacement assay parameters derived from Figure 5.

Surface Epitope	190 (●)	396 (□)	801 (▲)
Particle number (×10^−10^ mol·L^−1^)	5.3	2.6	1.3
Molar concentration of epitope (×10^−7^ mol·L^−1^)	1.0	1.0	1.0
D_50_ (µmol·L^−1^)	0.5	0.7	0.7
Limit of Detection (µmol·L^−1^)	0.01	0.01	0.01
Dynamic range (µmol·L^−1^)	0.01–39.8	0.01–39.8	0.01–39.8
